# Disparities in food allergy amongst British South Asian adult patients in central England^☆^

**DOI:** 10.1016/j.waojou.2025.101099

**Published:** 2025-09-05

**Authors:** Toni Osborne, Gareth Walters, Richard Baretto, Mamidipudi Thirumala Krishna

**Affiliations:** a Department of Allergy and Immunology, Birmingham Heartlands Hospital, University Hospitals Birmingham NHS Foundation Trust, UK; b Department of Immunology and Immunotherapy, School of Infection, Inflammation and Immunology, College of Medicine and Health, University of Birmingham, UK; c Department of Occupational Respiratory Medicine, Birmingham Heartlands Hospital, University Hospitals Birmingham NHS Foundation Trust, UK

**Keywords:** Food Allergy, South Asians, Asthma, Eczema, British, Disparities

## Abstract

**Background:**

Published evidence suggests an increased burden of food allergy (FA) amongst ethnic minority groups resident the United States of America and Australia, with limited data from the United Kingdom. The West Midlands Regional Allergy Service serves British Caucasian White and Ethnic Minority Groups with a large proportion being British South Asian, making it important to explore ethnicity—based differences in clinical outcomes for FA.

**Aims:**

To compare clinical outcomes of FA and other atopic diseases between Caucasian White and South Asian patients attending the regional allergy service in West Midlands.

**Methods:**

This prospective cross-sectional, observational study (N = 29 White and N = 21 South Asian) used a structured questionnaire with dichotomous, multiple-choice, and scaled questions to gather data on age, ethnicity, FA (including allergens, allergic reactions, adrenaline auto-injector use, and emergency department visits), and other atopic conditions.

**Main results:**

1.) South Asians had significantly (p = 0.006) more frequent FA reactions. 2.) Poorly controlled eczema and asthma were significantly (eczema p = 0.015, asthma p = 0.022) more common amongst South Asians. 3.) The burden of asthma, eczema, and allergic rhinitis was similar between groups. 4.) Significantly more White patients (p = 0.027) with asthma were on higher treatment steps.

**Conclusion:**

British South Asian patients with FA attending the West Midlands regional allergy service had more frequent allergic reactions and poorly controlled asthma and eczema compared to British Caucasian White patients. This highlights the need for improved education and compliance. Larger multi-centre studies are needed to gain further insight into ethnicity-based disparities in FA.

**Summary box**

• British South Asian (SA) patients with food allergy reported a higher rate of allergic reactions
• British Caucasian White patients with food allergy reported better control of asthma and eczema than SA patients

Healthcare inequities and inequalities is a major challenge for health services in High-Income Countries (HICs).^1^^,^^2^ We reported a higher incident risk of allergic rhinitis, asthma and atopic dermatitis amongst British South Asians (SAs), Afro-Caribbeans, and those of mixed ethnicity compared to the Caucasian White population.^3^ We and others also reported an enhanced risk of dust mite sensitisation and poor clinical outcomes from asthma and a higher incident rate of anaphylaxis amongst the British SAs.^4^, ^5^, ^6^ Data from Australia and United States suggest a higher prevalence of food allergy and food-induced anaphylaxis amongst Asian children.^7^^,^^8^ There are no published data regarding food allergy and associated comorbidity amongst British ethnic minority groups.

A cross sectional prospective observational pilot study (01 July 24 to 30 November 24) was conducted at the regional Allergy and Immunology Department, West Midlands to compare the clinical outcomes in food allergy and allergic comorbidities between British SA (Indian, Pakistani, Bangladeshi, and Sri Lankan) and Caucasian White adult patients (≥18 years). This centre receives referrals from a wide catchment area including West Midlands, Coventry and Warwickshire, Staffordshire, and Shropshire (https://geoportal.statistics.gov.uk/documents/9c17dbc2ea9e4d229ebf25337f9daa3c/explore). All patients had a formal diagnosis of type-1 hypersensitivity to food and attended clinic for a follow up appointment after having undergone a detailed allergy specialist evaluation with a systematic clinical history, skin prick tests and/or serum specific IgE (and component resolved diagnostic tests when deemed necessary) and were counselled regarding self-management of allergic reactions.

This study was approved by Health Research Authority and Health and Care Research Wales Research Ethics Committee and University of Southampton Ethics and Research Governance. Patients were interviewed by a single operator after seeking an informed consent and data were captured on a standardised paper-based study questionnaire. The questionnaire included age, gender, ethnicity, food allergy (allergens, number of reactions during the preceding 12 months, use of adrenaline autoinjector and emergency room visits), co-morbidities including asthma (exacerbations, asthma control test, treatment), hay fever, eczema and its severity, and whether or not patients benefitted from Allergy specialist evaluation. Demographic and outcome data variables were grouped into multiple categories (binary or more) and imported into Statistical Package for Social Sciences (SPSS, version 26; IBM 2020) for analysis. Data were summarised using frequency and proportion, and hypothesis testing undertaken at the 95% confidence level using Chi-squared tests. Data is summarised in Table-1.

**Table 1:** Summary of data

		British White Caucasian	British South Asian	P value
		N (%)	N (%)	
Age	18–25 years	15 (51.7%)	12 (57.1%)	p = 0.716
	26–35 years	10 (34.5%)	6 (28.6%)	
	36–45 years	3 (10.3%)	1 (4.8%)	
	46–55 years	0	1 (4.8%)	
	≥56 years	1 (3.3%)	1 (4.8%)	
Gender	Male	6 (20.7%)	8 (38.1%)	p = 0.176
	Female	23 (79.3%)	13 (61.9)	
	Non-binary	0	0	
	Prefer not to disclose	0	0	
Ethnicity		100%	100%	–
Generation (for South-Asians)	First generation	N/A	2 (9.5%)	
	Second generation	N/A	12 (57.1%)	
	Third generation	N/A	5 (23.8%)	
	More than third generation	N/A	2 (9.5%)	
How many foods are you allergic to? (Peanuts and tree nuts combined)	1 food	19 (65.5%)	9 (42.9%)	p = 0.119
	2-3 foods	9 (31%)	8 (38.1%)	
	>3 foods	1 (3.4%)	4 (19%)	
Which foods are you allergic to?	Fish/shellfish	5 (8%)	3 (5.3%)	–
	Tree nuts	23 (37%)	18 (32.1%)	
	Peanuts	20 (32.3%)	12 (21.4%)	
	Fruit	1 (1.6%)	3 (5.3%)	
	Legumes (excluding peanut)	5 (8%)	3 (5.3%)	
	Seeds	3 (4.8%)	7 (12.5%)	
	Egg	3 (4.8%)	8 (14.2%)	
	Milk	2 (3.2%)	2 (3.5%)	
Have you been provided with a clear advice to avoid food/s that you are allergic to?	Yes	25 (86.2%)	20 (95.2%)	p = 0.111
	To some extent	4 (13.8)	0 (0%)	
	No	0 (0%)	1 (4.8%)	
Have you been provided with clear verbal and written information about management of allergic reactions?	Yes	22 (75.9%)	15 (71.4%)	p = 0.882
	To some extent	4 (13.8%)	2 (9.5%)	
	No	3 (10.3%)	4 (19%)	
Do you see any challenges in management of your food allergy?	Yes	11 (37.9%)	10 (47.6%)	p = 0.617
	To some extent	9 (31%)	4 (19%)	
	No	9 (31%)	7 (33.3%)	
In the past 12 months, how many reactions have you had due to your food allergy?	0	16 (55.2%)	4 (19%)	p = 0.006
	1–3	13 (44.8%)	13 (61.9%)	
	>3	0 (0%)	4 (19%)	
In the past 12 months, how often have you used your adrenaline auto injector (AAI) i.e., EpiPen?	0	28 (96.6%)	17 (81%)	p = 0.174
	1–3	1 (3.4%)	3 (14.3%)	
	>3	0 (0%)	1(4.8%)	
In the past 12 months, how often have you attended the emergency department because of having an allergic reaction to food?	0	26 (89.7%)	16 (76.2%)	p = 0.316
	1–3	3 (10.3%)	4 (19%)	
	>3	0 (0%)	1 (4.8%)	
Eczema	Yes	17 (58.6%)	15 (71.4%)	P = 0.352
	No	12 (41.4%)	6 (28.6%)	
Hayfever	Yes	23 (79.3%)	18 (85.7%)	P = 0.561
	No	6 (20.7%)	3 (14.3%)	
Asthma	Yes	15 (51.7%)	13 (61.9%)	P = 0.474
	No	24 (48.3%)	8 (38.1%)	
From your point of view, how well is your eczema controlled?	Well controlled	11 (64.7%)	3 (20%)	p = 0.015
	Moderately controlled	5 (29.4%)	12 (80%)	
	Poorly controlled	1 (5.9%)	0 (0%)	
From your point of view, how well is your asthma controlled?	Well controlled	13 (86.7%)	6 (46.2%)	P = 0.022
	Moderately controlled	2 (13.3%)	7 (53.8%)	
	Poorly controlled	0 (0%)	0 (0%)	
ACT score	5–14	0 (0%)	3 (23.1%)	p = 0.048
	15–19	3 (20%)	0 (0%)	
	20–25	12 (80%)	10 (76.9%)	
In the past 12 months, how often have you experienced an exacerbation of your asthma symptoms?	0	8 (53.3%)	8 (61.5%)	p = 0.225
	1–3	4 (26.7%)	5 (38.5%)	
	>3	3 (20%)	0 (0%)	
Have you required oral steroid treatment for your asthma in the past 12 months?	Yes	2 (13.3%)	2 (15.4%)	P = 0.877
	No	13 (86.7)	11 (84.6%)	
British Thoracic Society treatment step		Caucasian White n (%) (n = 14)	South Asian N (%) (n = 10)	p = 0.027
	Step 1	5 (36)	3 (30)	
	Step 2	1 (7)	6 (60)	
	Step 3	0 (0)	0 (0)	
	Step 4	7 (50)	1 (10)	
	Step 5	1 (7)	0 (0)	

Sequential patients with established type-1 hypersensitivity to foods were approached with some declining participation; exact response rate was not captured in the study. Fifty patients were recruited (29 British Caucasian White and 21 SA). The 2 groups were comparable with respect to age and gender, 47 out of 50 were less than 45 years old, and there was predominance of female patients. A higher rate of food-induced allergic reactions was seen amongst the SA patients (81% vs 45%; p = 0.01), majority deemed mild-moderate. Ninety-seven percent and 81% of Caucasian White and SA patients, respectively, did not need to use their adrenaline autoinjector and there was no significant difference between the 2 groups. Fifty-five percent and 19% of Caucasian White and SA patients respectively did not report allergic reaction/s due to accidental exposure to food allergens. Forty-five percent of the Caucasian White patients reported 1–3 reactions in the preceding 12 months compared to 62% and 19% of SA patients who reported 1–3 and >3 reactions respectively. There was a broad representation of common food allergens between the 2 groups, although the proportion of patients with an allergy to egg and milk were greater amongst SAs. Seventy-two percent of patients in both groups reported allergic reactions to ≥2 food allergens. A significantly higher proportion of Caucasian White patients reported well controlled asthma (p = 0.02) and eczema (p = 0.02), although no significant differences were detected in frequency of asthma exacerbations and oral corticosteroid use between the 2 groups.

This is the first British study reporting poorer clinical outcomes in food allergy associated with a greater burden of uncontrolled asthma and eczema amongst British SA patients. The reasons underpinning disparities in clinical outcomes in allergic conditions amongst immigrant non-White populations resident in HICs has not been elucidated. This has however been attributed to multiple factors including deprivation, access to healthcare, poor compliance to treatment and self-management plans, as well as barriers related to language, healthcare access, and health literacy as well as cultural, social and religious factors.^1^^,^^2^

This study has limitations. First, the sample size is relatively small. The authors set out to achieve a total sample size of 90 (45 patients per group) as a part of a Master's project; however, due to practical and logistical challenges and time constrains, the sample size was limited to 50 patients. Second, the majority of patients was second or third generation, and there were limited opportunities to study first generation SA patients with a language barrier. Third, data relating to Index of Multiple Deprivation were not available. Fourth, the severity of eczema and asthma were assessed semi-quantitatively, however, this aligns with previous published reports of poor clinical outcomes amongst SAs.^4^^,^^5^

This study has highlighted a need for large multi-centre studies to gain deeper insight into ethnicity-based disparities in food allergy in the United Kingdom. A multipronged approach is needed employing qualitative and quantitative methodologies to gain deeper insight into understanding facilitators and barriers for allergy management, pattern of allergen sensitisation as well as severity of co-morbid conditions amongst British ethnic minority groups in order to pave the way for culturally tailored and culturally targeted supportive interventions, provision of a holistic clinical care, enhance patient engagement with health services as well development of novel primary and secondary prevention strategies.

## Availability of data and materials

Anonymised/pseudonymised dataset may be available upon request subject to approval/permission from University Hospitals Birmingham NHS Foundation Trust.

## Ethics approval

This study was approved by Health Research Authority and Health and Care Research Wales Research Ethics Committee and University of Southampton Ethics and Research Governance.

## Consent for publication

The authors’ consent for publication.

## Funding

None.

## Declaration of competing interest

MTK received research grants from NIHR for The SPACE study, The FAIR study and The TEAMS study outside the work presented in this manuscript. The data presented in this manuscript is TO's MSc Allergy project (University of Southampton).
